# Cannabinoid extract in microdoses ameliorates mnemonic and nonmnemonic Alzheimer’s disease symptoms: a case report

**DOI:** 10.1186/s13256-022-03457-w

**Published:** 2022-07-12

**Authors:** Ana Carolina Ruver-Martins, Maíra Assunção Bicca, Fabiano Soares de Araujo, Beatriz Helena Lameiro de Noronha Sales Maia, Fabrício Alano Pamplona, Elton Gomes da Silva, Francisney Pinto Nascimento

**Affiliations:** 1 Laboratório de Cannabis Medicinal e Ciência Psicodélica, Department of Medicine, Universidade Federal da Integração Latino-Americana, UNILA, Avenida Tarquínio Joslin dos Santos, 1000, Jardim Universitário I, Foz do Iguaçu, PR Brazil; 2 Department of Chemistry, Universidade Federal do Paraná, Curitiba, PR Brazil; 3 Department of Neurosurgery and Neurosciences, Johns Hopkins University, Baltimore, MD USA

**Keywords:** Alzheimer’s disease, Cannabinoids, Microdosing, Cannabinoid extract, AD treatment, Case report

## Abstract

**Background:**

Cannabinoid-based therapy has been shown to be promising and is emerging as crucial for the treatment of cognitive deficits, mental illnesses, and many diseases considered incurable. There is a need to find an appropriate therapy for Alzheimer’s disease, and cannabinoid-based therapy appears to be a feasible possibility.

**Case presentation:**

This report addresses the beneficial effect of cannabinoids in microdoses on improving memory and brain functions of a patient with mild-stage Alzheimer’s disease. The patient is a 75-year-old white man presenting with main symptoms of memory deficit, spatial and temporal disorientation, and limited daily activity. The experimental therapeutic intervention was carried out for 22 months with microdoses of a cannabis extract containing cannabinoids. Clinical evaluations using Mini-Mental State Examination and Alzheimer’s Disease Assessment Scale-Cognitive Subscale were performed.

**Conclusions:**

Here we provide original evidence that cannabinoid microdosing could be effective as an Alzheimer’s disease treatment while preventing major side effects. This is an important step toward dissociating cannabinoids’ health-improving effects from potential narcotic-related limitations.

## Background

Alzheimer’s disease (AD) is the most prevalent neurodegenerative disease among the elderly. Aging is the main risk factor, and since scientific medical advances are leading to longer life expectancy, experts expect AD to be the next global epidemic by 2050 [1]. Despite the latest medical and scientific advances, there is no efficient treatment to attenuate disease progression [2]. Some researchers consider that AD cannot be fully prevented, slowed down, properly diagnosed, or cured [1]. Currently, there are two categories of medication approved for AD palliative treatment, which slow degree disease progression to some degree but do not cure the disease: acetylcholinesterase inhibitors and *N*-methyl-d-aspartate (NMDA) blockers [3].

Besides neurochemical dysfunction (for example, cholinergic, glutamatergic), gliosis, neuroinflammation, oxidative stress, insulin resistance, and autophagy are well-described AD-associated phenomena. These events can be triggered or potentiated by Aβ and tau accumulation, the two major features of AD neuropathology [4]. Interestingly, antiinflammatory, pro-apoptotic, and antioxidant activities, as well as neurotropic and neurogenic stimulation, have been shown to be mediated by the endocannabinoid system [5]. Growing evidence suggests that there is endocannabinoid system dysfunction during AD progression [6–8].

The endocannabinoid system consists of endocannabinoid molecules, enzymes, and CB1R and CB2R (G_i_-coupled) receptors. In the brain, CB1R are mainly expressed in neurons regulating neurotransmitter release, while CB2R are expressed in immune cells (for example, T cells and microglia) reducing inflammation [9]. Naturally, the endocannabinoid system is the site of action for phytocannabinoids. Over 100 phytocannabinoids have already been identified in the *Cannabis sativa* plant, the most studied being tetrahydrocannabinol (THC) and cannabidiol (CBD). THC regulates synaptic transmission and promotes neuroprotection, acting as a CB1R and CB2R agonist [10], also known for its psychoactive and potent analgesic effects [11]. CBD inhibits endocannabinoid degradation/uptake and participates in CBR allosteric modulation, also known for its anticonvulsant and anxiolytic effects [11–13]. Typical THC-related adverse effects, namely intoxication, sedation, and tachycardia [14], are mitigated by combination of both phytocannabinoids.

The main premise of this study is that phytocannabinoids administered in microdoses can mitigate AD-induced neurochemical imbalance. Of note, an extensive preclinical and clinical review demonstrated the therapeutic use of cannabinoids for panoply disease, including AD [15]. In addition, a synthetic cannabinoid agonist (0.5 mg) has shown beneficial effects on AD-related aggressiveness and night mood swings, for at least 3 months [16]. Our hypothesis is supported by many animal studies [10] but has been unclear in AD human studies [17]. Herein, we describe the beneficial effect of an orally administered phytocannabinoids extract (8:1; THC:CBD ratio) on mnemonic and nonmnemonic symptoms in one patients with AD, as evaluated by Mini-Mental State Examination (MMSE) and Alzheimer’s Disease Assessment Scale-Cognitive Subscale (ADAS-Cog). We originally report initial but fundamental evidence that chronic cannabinoid microdosing successfully treated a patient with AD, using less than 1 mg of THC per day, for long-term effectiveness and sustainable quality of life.

## Case presentation

### Patient history

The patient is a 75-year old white man of italian descent, married, with three children and five grandchildren. He is an autonomous farmer, countryside resident of Planalto, Paraná, Brazil, a city with roughly 14,000 people, where he participates in flea markets, church groups, and farming negotiations. He quit smoking 1 year before the start of this experimental treatment, after 45 years of daily cigarette consumption, and had no history of alcohol overconsumption. Besides that, the patient was overall healthy, with no diagnosed comorbidity of any nature, cardiovascular, kidney or hepatic dysfunction/disease, dyslipidemia, diabetes mellitus, or any other neurological diseases beyond AD. Hence, he was using no other continuous medication.

General physical examination remained unchanged while monitored throughout the entire experimental treatment, including pulse, blood oxygenation, and pressure. Further, blood work was periodically requested to assess renal, hepatic, and hemostatic functions, as well as lipidic and glucose panels. Patient showed average heart rate of 83 beats per minute and blood pressure of 112 over 76 mmHg. A summary of general good health condition, based on blood work history, is presented in Table 1.

**Table 1:** Summary of laboratory tests throughout the experimental treatment and follow-up

Laboratory test		Reference value	24 April 2017	8 November 2017	9 September 2019	11 April 2020	26 March 2022
			Result	Result	Result	Result	Result
Urea		18–55 mg/dL	31 mg/dL			30.8 mg/dL	26.8 mg/dL
Urinalysis					Normal		Normal
Comprehensive metabolic panel	Creatinine	0.5–1.3 mg/dL	1.06 mg/dL			0.9 mg/dL	1.08 mg/dL
	Glucose	66–99 mg/dL	88 mg/dL	95 mg/dL	70 mg/dL		103.7 mg/dL
	Aspartate amino transferase	< 40 U/L	11.4 U/L			12.8 U/L	16.3 U/L
	Alanine amino transferase	<38 U/L	9.2 U/L			7.4 U/L	14 U/L
CBC with differential	Red blood cell count	4.50–5.9 million/mm^3^	4.8 million/mm^3^		4.75 million/mm^3^	4.47 million/mm^3^	4.53 million/mm^3^
	Hemoglobin	13.5–17.5 g/dL	13.5 g/dL		14.58 g/dL	14 g/dL	14 g/dL
	Hematocrit	41–53 %	40.70%		45%	40.80%	43.40%
	Platelet count	140,000–450,000/mm^3^	183,000/mm^3^		240,800/mm^3^	225,000/mm^3^	234,000/mm^3^
	White blood cell count	5000–10,000/mm^3^	9200/mm^3^		9000/mm^3^	7400/mm^3^	7500/mm^3^
TSH		0.34–5.60 µ[IU]/mL	1.58 µ[IU]/mL			1.64 µ[IU]/mL	
Lipid panel	Triglycerides	Up to 150 mg/dL	122.6 mg/dL	97 mg/dL	146 mg/dL		153.2 mg/dL
	HDL	> 60 mg/dL	44 mg/dL	46 mg/dL			
	LDL	< 100 mg/dL	221 mg/dL				
	Total cholesterol	Up to 200 mg/dL	197 mg/dL	198 mg/dL	196 mg/dL		165 mg/dL

*CBC* complete blood count,* TSH* thyroid-stimulating hormone,* HDL* high-density lipoprotein,* LDL* lowdensity lipoprotein

### AD history

The patient was diagnosed with AD 2 years prior to the start of this experimental treatment, according to brain magnetic resonance imaging, anamnesis, and clinical assessment, which includes and is not limited to the use of National Institute of Neurologic and Communicative Disorders and Stroke, and the Alzheimer Disease and Related Disorders Association (NINCDS-ADRDA) criteria. Our neurologist had access to the transcripts of his imaging examinations, which revealed no significant alterations and ruled out other possible causes of dementia, namely cerebrovascular disease or stroke, frontotemporal alterations, and/or evidence of other neurological disorders.

Specifically, patient was diagnosed with AD stage 1 and 4 according to the Clinical Dementia Rating (CDR) and the Global Deterioration Scale/Functional Staging of Dementia the Alzheimer Type (GDS/FAST), respectively. Clinical neurological conditions showed memory loss; spatial and temporal disorientation; forgetfulness (for example, regarding people and facts), constant storytelling on repeat mode; lack of initiative; signs of possible depression; struggling with organization, planning, and executing actions; incapability of performing simple hygiene and cooking tasks; and, thus, inability to live unassisted.

Memantine (10 mg/day orally) was the current undergoing treatment, though he experienced rapid disease progression (decreased cognitive function) and adverse effects (mostly dizziness followed by falls, headache, and constipation). Following the neurologist’s recommendation, treatment with memantine was suspended, given the lack of efficacy and the above-mentioned rapid symptomatic progression.

### Experimental AD treatment with cannabinoid microdosing

This experimental design was a pharmacological intervention using cannabinoid extract followed by mnemonic and nonmnemonic symptom assessment over the course of 22 months, conducted by the neurologist integrating our research group. It was conducted in accordance with the Declaration of Helsinki on Ethical Principles for Medical Research Involving Human Subjects, adopted by the General Assembly of the World Medical Association (1996), and followed the Brazilian Health Ministry (from Portuguese, *Ministério da Saúde do Brasil*) recommendations. The ethical committee of Unioeste University under number 2788021 approved this case report, and the patient provided written informed consent to publish this case report.

**Fig. 1:**
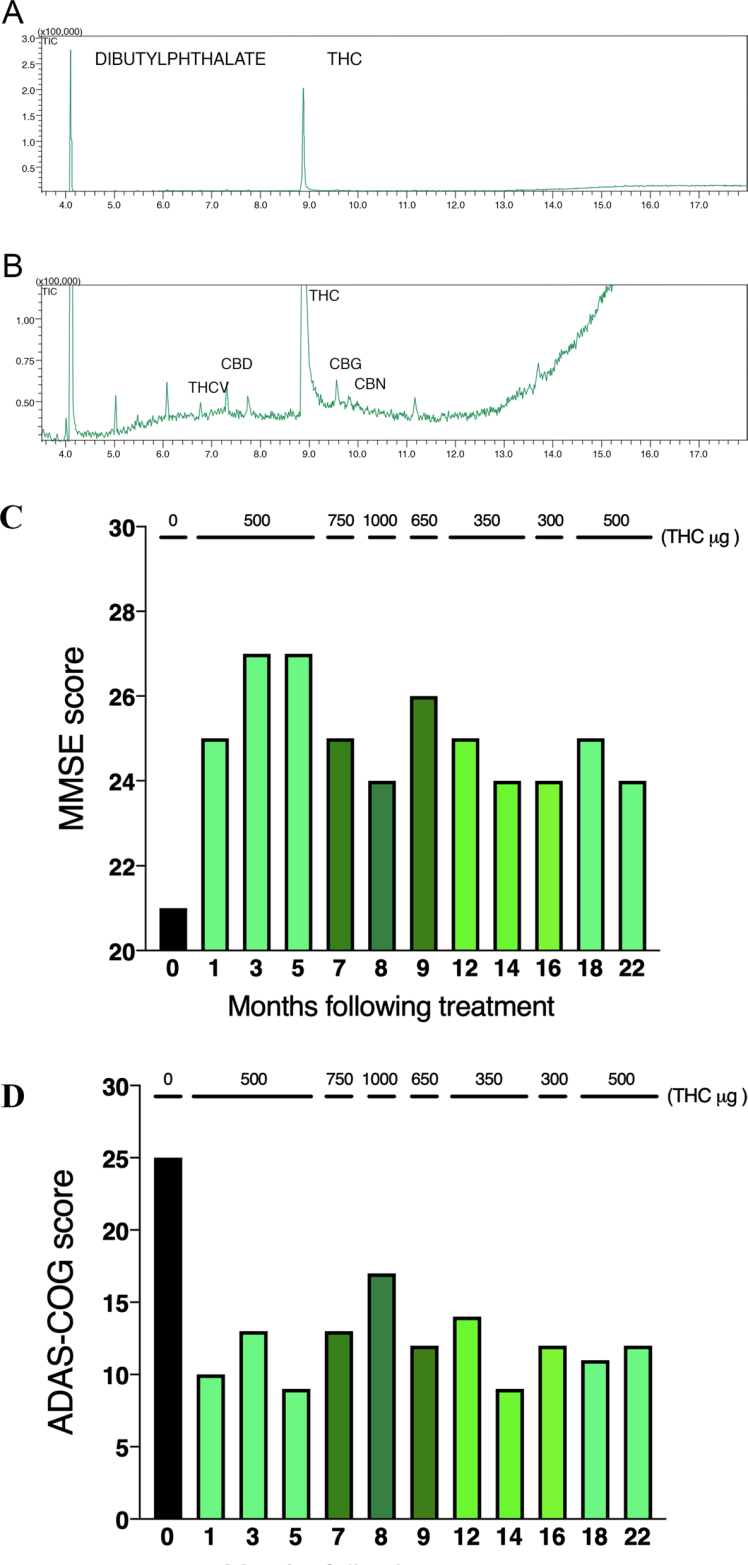
Chemical analyses of the THC-rich cannabis extract and patient’s clinical cognitive evaluation timeline. **A** The extract analyzed has a higher prevalence of THC (8.7 ± 0.5% m/m) and lower concentration (0.75 ± 0.15%) of CBD. **B** A lower prevalence of other phytocannabinoids was detected. **C**, **D** Scores according to the Alzheimer’s Mini-Mental State Examination (MMSE) (**C**) and the Alzheimer’s Disease Assessment Scale-Cognitive (ADAS-Cog) scale (**D**). Bars represent different THC microdosages (µg). Dose distribution was the following: 500 µg for the initial 150 days of treatment, 750 µg for the following 60 days, 1000 µg for the next 30 days, 650 µg during the next 30 days, 350 µg for the following 60 days, 300 µg THC for 30 days, and 500 µg for the last 60 days of treatment. T0 (black bar), baseline assessment before the start of experimental treatment; T1 to T22 (bars colored in shades of green), treatment with cannabinoid extract, in which shades correspond to doses of THC, from lighter (lowest dose) to darker (highest dose)

The patient’s family imported the cannabis extract as a dietary supplement and approached the university for clinical anamnesis and a proper extract analysis, since THC and CBD doses are not tested in dietary supplements. As previously described at American Herbal Pharmacopoeia [18], we used gas chromatography—mass spectrometry (GC–MS) to determine the major cannabinoid dosage in the extract. The THC:CBD ratio was 8:1, henceforth referred to as “cannabinoid extract” (Fig. 1A and B).

Interventions with the cannabinoid extract started after a baseline assessment (T0) and are expressed in the graph as its equivalent dose of THC. Initial treatment consisted of 500 µg THC for the initial 150 days; 750 µg THC during the next 60 days; 1 mg THC for 30 days; 650 µg THC on the following 30 days; 350 µg for 60 days; 300 µg THC for 30 days; and finally, 500 µg of THC for 60 days. The dose fluctuation reflects the clinical decisions in the attempt of finding an optimal dose, 500 µg being the most used dose. Of note, the patient continues to use the extract at this dose without any additional drug of continuous use, after the official evaluation/follow-up for this case report ended.

The Mini-Mental State Examination (MMSE) and the Alzheimer’s Disease Assessment Scale (ADAS-Cog) were the scales utilized for patient evaluation and data acquisition. The former is widely employed in several neurophysiology evaluations and epidemiologic studies. It is a useful tool for the assessment of time and spatial orientation, memory, calculus capacity, language, identification patterns, comprehension, writing skills, and drawing [19, 20]. Low scores indicate negatively affected brain function that could be associated with AD. However, it should not be used as the only diagnostic tool. The latter comprises a series of assessments created to evaluate the cognitive function of patients with AD. The ADAS-Cog is composed of 11 tests assigned to evaluate memory, orientation, language, praxis, and other cognitive capabilities [21], in which a high score indicates high disease severity. It is one of the most frequently utilized tests to assess cognition in research studies and clinical trials for new drugs and other interventions.

The scale-oriented evaluation was carried out on day T0 (previous to treatment) and days 30 (T1), 90 (T2), 150 (T3), 210 (T4), 240 (T5), 270 (T6), 360 (T7), 420 (T8), 480 (T9), 540 (T10), and 660 (T11) following the start of the treatment. Unfortunately, we did not apply standardized scales for psychiatric assessment of mood, anxiety, and sleep quality, which were the variables qualitatively assessed using patient and caregiver testimonials, recorded at each evaluation.

Here we report evidence that the cannabinoid extract improved MMSE (Fig. 1C) and ADAS-Cog (Fig. 1D) scores in the subject evaluated. Symptom amelioration was rapid, robust, and not limited to mnemonic. We tried to titrate the dose up to 1 mg THC, but the most frequent dose was 500 µg THC. The period when the patient was treated with this dose seemed to be the period with higher symptom suppression. This well-known cannabinoid bell-shaped effect was not surprising since it has been previously reported [22–25].

Additionally, testimonials from patient and caregiver highlight other cognitive improvements. As described by the patient himself, “*I used to feel forgetful, not once after the treatment. Sometimes, I did not know where I was, it has not happened to me anymore. I used to find myself lost on the streets, I could not leave home unassisted; today, I took the bus by myself to perform my clinical evaluation*.” Of note, the treatment with the cannabinoid extract in microdoses appears to positively affect not only cognitive functions. Likewise, the patient has described other enhancements: “*Shortly after the beginning of the treatment, I already felt more alert and excited during daily activities, and I have noticed I have been sleeping much better*.”

The treatment here described mitigated AD symptoms, with rapid onset and long-term consequences. In this report, cognitive and memory enhancement lasted for more than 1 year following the start of treatment, and remained stable while we progressively evaluate/follow up with the patient, for more than 1 year after the official report ended. At that point, 42 months after using the cannabinoid extract, the cognitive assessment showed an MMSE score of 24 and an ADAS-Cog score of 10, demonstrating that our patient was still stable. Imaging tests, including computed tomography (ruling out other probable causes of dementia), neurological examination, serum tests for thyroid, kidney, liver, electrolytes, and complete blood count were all normal before the start of the experimental treatment and remained unchanged throughout the patient’s follow-up period (Table 1).

## Discussion and conclusion

This case report describes the therapeutic effect of cannabinoids microdosing using a THC-rich extract for the treatment of mnemonic and nonmnemonic symptoms of a patient with AD. The treatment induced an increase of Mini-Mental State Examination (MMSE) and a reduction of Alzheimer’s Disease Assessment Scale-Cognitive Subscale (ADAS-Cog) scores. In addition, the patient and his caregiver have reported a substantial improvement in quality of life, while further behavioral and biochemical follow-up evaluations showed no signs of toxicity or significant side effects. This experimental treatment represents an improvement compared with current approved Alzheimer’s treatment that slows disease progression for a short period of time [26, 27]. Also, a possible advance compared with previous literature on cannabinoid use for other neurological diseases, using much higher doses [28, 29].

It is remarkable how a dose so significantly lower than those previously reported is able to consistently improve cognitive and noncognitive AD symptoms. For instance, Sativex is normally administered up to 20 mg of THC per day [30, 31], while the dosage here never exceeds 1 mg of THC per day. The importance of using preparations with controlled composition, as well as the careful selection of dose and route of administration for cannabinoid extracts, was previously discussed [15]. We emphasize this drastic difference in dose because we used cannabinoids with a microgram range, suggesting this could be the main reason why symptoms improved while no noticeable side effects were observed.

Treatment revealed nonpredicted positive outcome on nonmnemonic previously described AD symptoms, such as mood swing, aggressiveness, and bipolar personality [32], which we consider essential for patient adherence to treatment. It is also conceivable to hypothesize that the patient’s better performance on MMSE/ADAS-Cog could be explained by the cannabinoid-induced improvement in psychological well-being (mood, sleep, and anxiety). However, given that the impressive improvement in the MMSE/ADAS-Cog could not be achieved solely with the use of antidepressants or anxiolytics (normally used as adjuvant therapy for AD), we consider those as secondary beneficial effects of the treatment. Still, we cannot discard the possibility that the effects of the extract are CBR-independent, since phytocannabinoids can also act on other G-protein-coupled receptors (GPCRs), transient receptor potencial channels (TRPs), and ion channels [33].

Although it is certainly far-fetched to speculate on the mechanisms behind our clinical observations, it might be conceivable that the primary beneficial effect on memory/cognition is provoked by compensatory low doses of THC for an aging-impaired endocannabinoid system. In fact, myriad papers have reported cannabinoid effects on AD using experimental *in vitro* and *in vivo* models. For instance, cannabinoid treatment attenuates Aβ and neurofibrillary tau accumulation, as well as memory deficits in AD transgenic mouse models [34, 35]; blocks Aβ neuronal proteolysis and prevents Aβ aggregation [36]; mitigates Aβ-induced neuroinflammation and oxidative stress [37]; whereas favoring neurogenesis factors [that is, brain-derived neurotrophic factor (BDNF)] and antiinflammatory cytokine release, as well as presynaptic and axonal proteins upregulation [10, 34, 37–45]. Thus, we are also hypothesizing that the long-term positive effects of the cannabinoid extract may be due to reduction in AD-related neuroinflammation.

Our results are unprecedented and very encouraging. However, we must consider the limitations of a one-patient case report, without blinding or placebo group. In addition, it would be important to use psychiatric scores for quantitative assessment of mood, anxiety, and sleep quality as well as to quantify inflammation- and AD-related biomarkers in blood and liquor, thus acquiring substantial data for better elucidating the cannabinoid extract mechanisms of action. We decided to follow up with this patient as a “typical case” to gain insight for a future clinical trial, addressing the above mentioned limitations, which is currently underway.

In summary, data presented in this case report suggest that cannabinoid microdosing is a potential therapeutic for AD, with no significant side effects, although placebo-controlled clinical trials are needed to confirm and extend these data.

## Data Availability

All data generated or analyzed during the evaluation of this experimental treatment are included in this published article.
